# Development of an annually updated Japanese national clinical database for chest surgery in 2014

**DOI:** 10.1007/s11748-016-0697-1

**Published:** 2016-08-08

**Authors:** Shunsuke Endo, Norihiko Ikeda, Takashi Kondo, Jun Nakajima, Haruhiko Kondo, Kohei Yokoi, Masayuki Chida, Masami Sato, Shinichi Toyooka, Koichi Yoshida, Yoshinori Okada, Yukio Sato, Meinoshin Okumura, Munetaka Masuda, Koji Chihara, Hiroaki Miyata

**Affiliations:** 1 Committee for NCD, The Japanese Association for Chest Surgery, 3F Chiyoda Seimei Kyoto Oike Building 200 Takamiya-cho, Takakura-Oike-dori, Nakagyo-ku, Kyoto 604-0835 Japan; 2 Committee for Scientific Affairs, The Japanese Association for Thoracic Surgery, 1F Teral Kohraku Building, 2-3-27 Kohraku, Bunkyo-ku, Tokyo 112-0004 Japan; 3 The Japanese Board of General Thoracic Surgery Tokyo, 1F Teral Kohraku Building, 2-3-27 Kohraku, Bunkyo-ku, Tokyo 112-0004 Japan; 4 The Japanese Board of General Thoracic Surgery Kyoto, 3F Chiyoda Seimei Kyoto Oike Building 200 Takamiya-cho, Takakura-Oike-dori, Nakagyo-ku, Kyoto, 604-0835 Japan; 5 National Clinical Database, 1-8-3 Marunouchi, Chiyoda-ku, Tokyo, Japan; 6 Department of Thoracic Surgery, Jichi Medical University, Tochigi, Japan; 7 Department of Thoracic Surgery, Tokyo Medical University Hospital, Tokyo, Japan; 8 Department of Thoracic Surgery, Tohoku Pharmaceutical University Hospital, Miyagi, Japan; 9 Department of Thoracic Surgery, University of Tokyo Graduate School of Medicine, Tokyo, Japan; 10 Department of General Thoracic Surgery, Kyorin University Hospital, Tokyo, Japan; 11 Department of Thoracic Surgery, Nagoya University Graduate School of Medicine, Aichi, Japan; 12 Department of General Thoracic Surgery, Dokkyo Medical University, Tochigi, Japan; 13 Department of General Thoracic Surgery, Graduate School of Medical and Dental Sciences, Kagoshima University, Kagoshima, Japan; 14 Department of Thoracic, Breast and Endocrinological Surgery, Graduate School of Medicine, Dentistry and Pharmaceutical Sciences, Okayama University, Okayama, Japan; 15 Department of Thoracic Surgery, Institute of Development, Aging and Cancer, Tohoku University, Miyagi, Japan; 16 Department of Thoracic Surgery, Faculty of Medicine, University of Tsukuba, Ibaraki, Japan; 17 Department of General Thoracic Surgery, Osaka University Graduate School of Medicine, Osaka, Japan; 18 Department of Surgery, Yokohama City University, Kanagawa, Japan; 19 Division of Thoracic Surgery, Shizuoka City Shizuoka Hospital, Shizuoka, Japan; 20 Department of Health Policy and Management School of Medicine, Keio University, Tokyo, Japan

**Keywords:** Chest surgery, Nationwide survey, Database, Internet, Board certification

## Abstract

**Objectives:**

A national clinical database (NCD) adopted an “Internet-based collection” in 2011. An NCD specializing in chest surgery was launched based on the NCD system in 2014. The system was linked to the board certification as the second level in the hierarchy of the specialty of chest surgery and accreditation of educational institutions for chest surgery. Here, we report the status of the NCD for chest surgery in 2014 and clarified its registration rate and its accuracy.

**Methods:**

Chest surgeries undertaken in Japan since January 1st, 2014 until the end of the same year were registered through an Internet-based system until April 8th, 2015. The registration rate was compared with the annual survey conducted by the Japanese Association for Thoracic Surgery (JATS) from 2011 to 2014. The rate of accurate inputting was measured by an Internet-based audit in reference to 563 anonymous operative notes of patients presented by 106 chest surgeons at the time of renewal for board certification for chest surgery.

**Results:**

A total of 88,112 chest-surgical procedures were registered from 1000 chest surgery units (CSUs). Distribution of procedures by thoracic disease was almost identical to that of the annual survey conducted by JATS. However, the NCD had 4260 more registered procedures compared with the annual survey. The Internet-based audit showed that inter-rater agreement between Internet-based data and operative notes in any item was >94 %.

**Conclusions:**

The NCD system can sustainably provide important and up-to-date information relating to preoperative status, oncology, and best practice for chest surgery in Japan.

## Introduction

Clinical databases can be evaluated according to the quantity/quality of data, “updatability” and database size. Two surveys for chest surgery in Japan have been conducted: an annual survey by the Japanese Association for Thoracic Surgery (JATS) [1] and one by the Japanese Joint Committee of Lung-Cancer Registry [2]. Both were performed by mail from the flagship CSUs. The former is a conventional annual survey collected by JATS since 1986 and linked to the accreditation of educational institutions for chest surgery, and therefore, the survey has a high-registration rate. It focused only on the number of surgical procedures and their 30-day mortality and hospital mortality; few data on patient characteristics and perioperative evaluations are available. The annual survey by the Japanese Joint Committee of Lung-Cancer Registry has been conducted irregularly four times since 1994 and has focused on primary lung cancer. The size of that database accounted for fewer than half of the surgical procedures undertaken at the respective year in Japan. The quantity and quality of data on the oncologic characteristics of this database were of such a high standard that they were submitted to the staging committee of the International Association for the Study of Lung Cancer, and had a considerable impact on the seventh edition of guidelines for the staging of lung cancer. Each database is good in some aspects, yet poor in others. An “ideal” database has yet to be created.

An NCD in Japan adopted a “web-based collection” in 2011 with significant support from the Japan Surgical Society (JSS) [3]. Nine other surgical societies (Japanese Society for Gastroenterological Surgery; Japanese Society for Cardiovascular Surgery; Japanese Society for Vascular Surgery; JATS; Japanese Association for Chest Surgery (JACS); Japanese Society for Pediatric Surgeons; Japanese Breast Cancer Society; Japan Association for Endocrine Surgeons; and Japanese Society of Thyroid Surgery) joined the NCD. The NCD is a nationwide collaboration associated with the Japanese Surgical Board Certification System, in which data on 1.6 million surgical procedures from >4000 hospitals were collected in 2014.

An NCD specializing in chest surgery based on the existing NCD system was launched in 2014. The NCD for chest surgery required more detailed clinical data in addition to basic data. The NCD is linked to the second level in the hierarchy of specialty chest surgery and accreditation of educational institutions for chest surgery through web-based conversion, both of which are supported by the Japanese Board of General Thoracic Surgery.

### NCD for chest surgery

#### Inclusion and exclusion criteria

Inclusion criteria of the NCD were surgeries via thoracotomy, thoracoscopy, or mediastinoscopy under general anesthesia. Exclusion criteria were: (1) surgery under local anesthesia; (2) bronchoscopic intervention with a tracheal stent; (3) percutaneous or endobronchial intervention in the thoracic cavity or mediastinum; and (4) removal of a stainless-steel metal bar after the Nuss procedure.

#### Data entry system

**Fig. 1:**
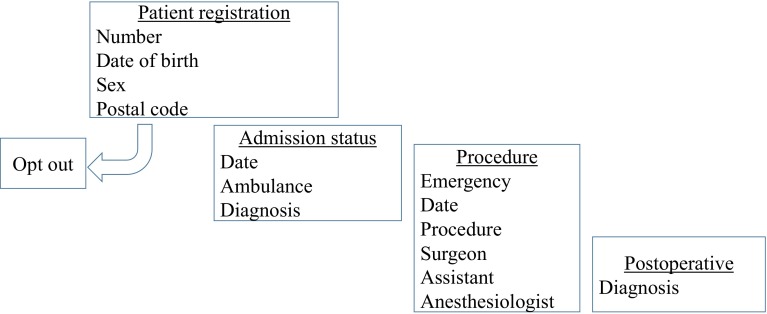
First step of NCD registration. The essential items required for the first step of NCD registration. The postal code of the patient must be inputted. Surgeons and assistants can be identified by the license number registered by the Japanese Surgical Society

Chest-surgical procedures undertaken in Japan since January 1st, 2014 until the end of the same year could be registered until April 8th, 2015. Data were registered after patient data had been anonymized. Submission of patient data to the NCD (except for presumably, the very few (if any) patients who wished to opt out) was a prerequisite for the Japanese Surgical Board Certification System. Essential data were inputted at the first step for registration (Fig. 1). Surgeon-related data (identified by the license number of each surgeon registered by the JSS) were related to the surgeon’s performance and referred to board certification at the first level of the hierarchy for surgery. The first step of registration could be completed if the operating surgeon did not switch to another field of surgery. The second step of registration started according to the field of surgery chosen as the second level of hierarchy. If “chest surgery” was selected as the second level of hierarchy, the detailed clinical data in relation to chest surgery were inputted until the completion of data registration without alerts showing inputting mistakes. Data were referred to board certification at the second level of surgery for chest surgery by the Japanese Board of General Thoracic Surgery and converted to annual survey data by JATS, if needed, which referred to the accreditation of educational institutes for chest surgery.

#### Data items for chest surgery

**Fig. 2:**
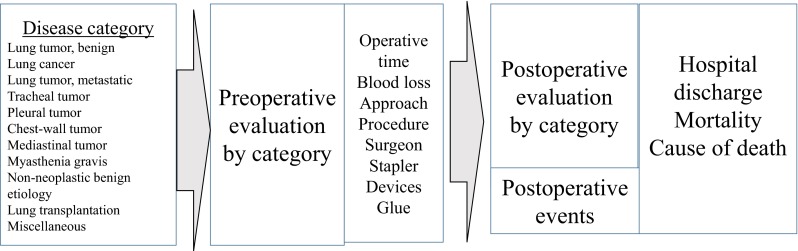
Chart of input items for perioperative information. In the first step of the registry process for chest surgery, the operation is categorized as a surgical thoracic disease. All input items, which are displayed according to the thoracic disease, must be filled out. Perioperative evaluations include common items and unique items according to the categorized disease. Finally, discharge information is inputted. These clinical data in relation to chest surgery were inputted until the completion of data registration without alerts showing inputting mistakes

For the initial step in the registration process for chest surgery, the procedure is categorized as a “surgical thoracic disease”, as a definitive diagnosis irrespective of the diagnosis upon hospital admission (Fig. 2). Disease category was identical to that for the annual survey by JATS, so that the NCD could be converted to the annual survey by JATS. All inputted items (which were displayed according to these thoracic diseases) had to be completed. Perioperative evaluations included “common” items and “unique” items according to the categorized disease (Tables 1, 2). Clinical and pathologic staging was inputted if the procedure was categorized as that for lung cancer. If procedure-related complications occurred within 30 days after surgery, postoperative-complication items were registered even after hospital discharge (Table 3). If redo-surgery was necessary before discharge, the second procedure was registered as “second surgery”. The patient was registered as “second admission and the following surgery” if the patient was discharged from hospital after the first surgical procedure and underwent redo-surgery. Finally, information relating to hospital discharge was inputted (Table 4). The outcome was classified as “in-hospital death” even if the patient died after transfer to another hospital. The cause of surgical procedure-related death (including 30-day mortality and in-hospital mortality) was identical to that used for the annual survey by JATS and had to be inputted (Table 5).

**Table 1:** Preoperative information

Height, weight and performance status
Pulmonary function test (VC*^1^, FVC*^2^, FEV1.0*^3^)
Smoking history and status
Anticancer treatment (if the disease is a thoracic malignancy)*^4^
Comorbidity
Coronary artery disease*^5^
Anticancer treatment within the past 5 years
Disease of the central nervous system*^6^
Diabetes mellitus*^7^, hemodialysis, anemia*^8^
Interstitial pneumonia*^9^, Liver failure*^10^
Autoimmune disease*^5^, arrhythmia*^6^, hypertension*^6^
Other (supplementary explanation)
Clinical TNM staging for lung cancer

^1^Vital capacity

^2^Forced vital capacity

^3^Forced expiratory volume in 1 s

^4^Chemotherapy, radiotherapy, chemoradiotherapy, other

^5^Required treatment or previous treatment

^6^Required treatment

^7^Required treatment and supplementary explanation

^8^Hemoglobin concentration ≤8.0 g/dL

^9^Interstitial pneumonia shadow on computed tomography of the chest

^10^Child–Turcotte classification B or C

**Table 2:** Operative information

Operative time, blood loss, transfusion, intraoperative accident
Approach (open, VATS, robot)
Procedure (main procedure, nodal dissection, combined resection)
Supplement
Number of cartilages stapled, energy device (except electric cautery), fibrin glue, extracorporeal life support
Lung cancer
Detailed VATS approach: number of access ports, maximum diameter of the wound
Histology, pathologic TNM staging
Thoracic neoplasm (other than lung cancer)
Histology
Pneumothorax
Surgical procedure, buttress sheet

**Table 3:** Postoperative events

Pulmonary
Prolonged air leak*^1^
Atelectasis*^2^
Pneumonia*^3^
Acute exacerbated interstitial pneumonia
Respiratory failure*^4^
ARDS*^5^
Bronchopleural fistulae
Pulmonary emboli
Cardiovascular
Arrhythmia*^6^
Myocardial infarction
Congestive heart failure*^6^
Neurology
Cerebral hemorrhage
Cerebral infarction
Hoarseness
Delirium*^6^
Infection
Empyema*^7^
Mediastinitis*^7^
Wound infection*^7^
Miscellaneous
Redo-surgery within 24 h
Bleeding*^8^
Chylothorax
Renal failure*^9^
Liver failure*^6^
Other (supplementary explanation)

^1^Air leak >6 days duration or required treatment

^2^Required bronchoscopy

^3^Infection signs and infiltration shadow on chest radiography

^4^Ventilation support >48 h

^5^Adult respiratory distress

^6^Required treatment

^7^Required drainage

^8^Required blood transfusion

^9^Required hemodialysis, postoperative serum concentration of creatinine >4 mg/dL or more than three times the preoperative value

**Table 4:** Discharge information

Date
Status upon hospital discharge (dead^a^ or alive or transfer to another hospital)
Status 30 days after surgery (dead or alive)
Redo-surgery

^a^Outcome was classified as “in-hospital death” even if the patient died after transfer to another hospital

**Table 5:** Cause of death

Primary disease
Other malignancy
Cardiovascular related
Cerebrovascular related
Pneumonia
Interstitial pneumonia
Empyema/mediastinitis
Bronchopleural fistulae
Respiratory failure
Pulmonary emboli
Other (supplementary explanation)
Uncertain

#### Data registration

To assure the traceability of data through an Internet-based data-management system, directors of CSUs who were authorized by the NCD office approved of their data being registered in departments in charge of annual cases and responsible for data entry. They could revise web-based data whenever they noticed errors, especially at the time of conversion to the form of the annual survey by JATS, until April 8th, 2015.

#### Fixed data from an NCD for chest surgery

The data were completely fixed at April 9th, 2015. From June onwards, data identified by the license number of the surgeon registered by the JSS could reflect the surgeon’s performance for board certification at the second level of surgery for chest surgery by the Japanese Board of General Thoracic Surgery. Data converted to the annual survey 2014 by JATS in 706 CSUs out of 737 CSUs were presented to reflect institutional accreditation of CSUs from June onwards. Thirty one CSUs presented data to the annual survey by mail as in the past.

## Methods

**Fig. 3:**
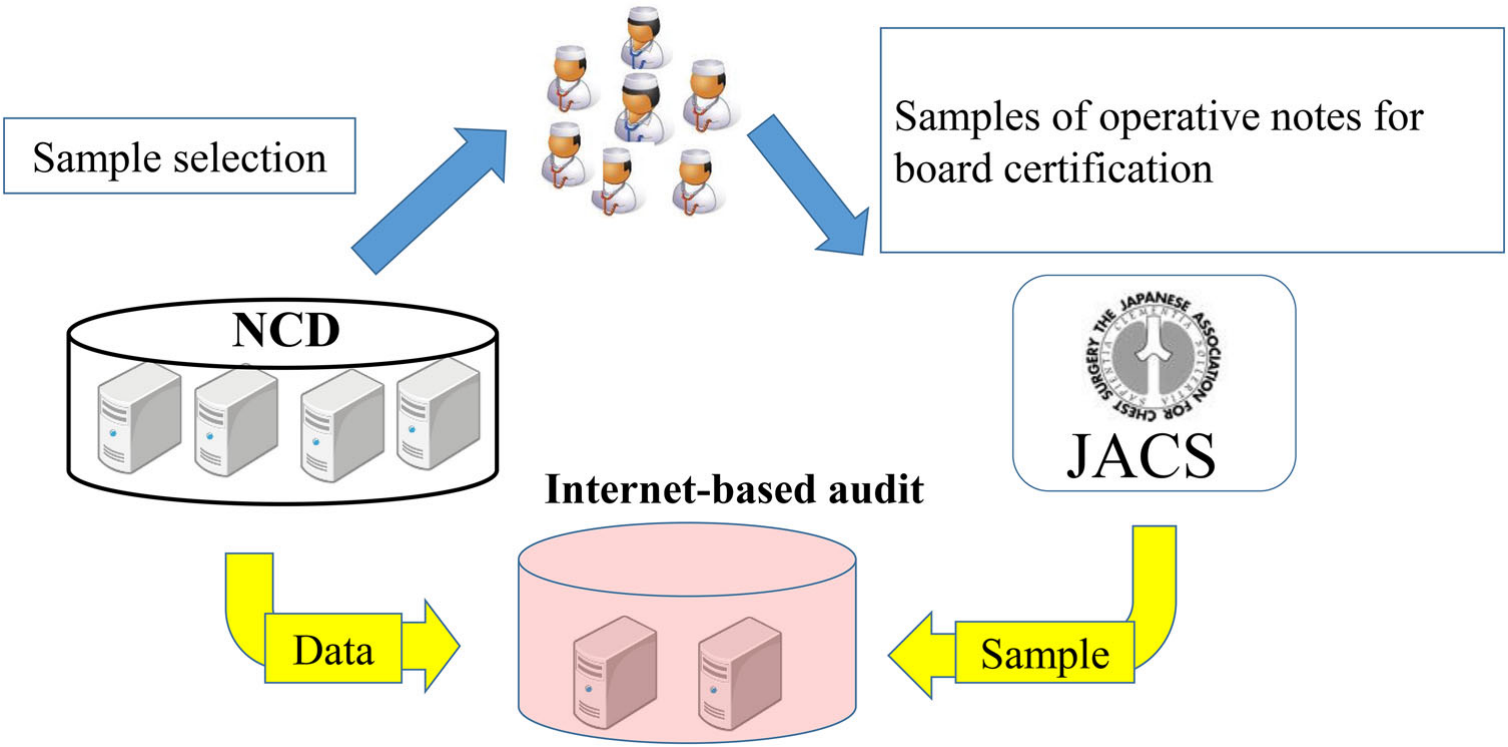
Internet-based audit system. An Internet-based audit system was developed to maintain the data quality of the NCD for chest surgery. Surgeons had to provide anonymous operative notes of patients selected randomly by the NCD and given to the JACS at the time of application for board certification for chest surgery. A committee authorized by the NCD and JACS could check the inter-rater reliability between these samples and Internet-based data from the NCD

The registration rate was compared with the annual survey between 2011 and 2014. Accurate input rate for certain items (age, sex, procedure, disease, operation time, blood loss, and surgeons) was measured by an Internet-based audit in reference to 563 anonymous operative notes of patients selected randomly by the NCD presented from 106 chest surgeons at the time of renewal for board certification for chest surgery. Authorized committees by the NCD and JACS could check inter-rater agreement between these operation notes and Internet-based data from the NCD office (Fig. 3).

## Results

**Fig. 4:**
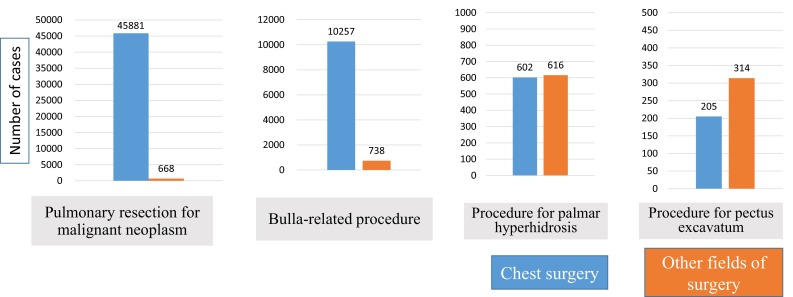
Number of patients registered for chest surgery and for other fields of surgery of the NCD 2014 for each surgical procedure. Malignant neoplasm included lung cancer and metastatic lung tumor. Bulla-related procedures included bullectomy and volume reduction surgery

**Fig. 5:**
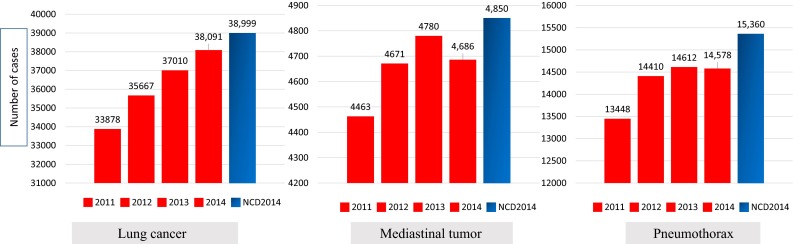
Comparison between the annual report by JATS and NCD 2014 shows the number of registrations to be almost identical for the selected thoracic diseases

**Fig. 6:**
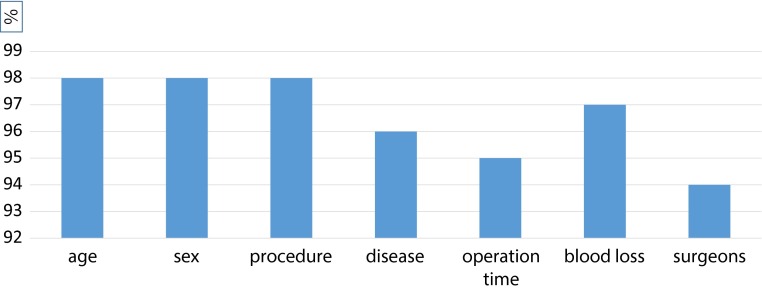
Ratio of inter-rater agreement of operative data based on 563 operative notes. Agreement was >94 % which is acceptable, but will improve with better education and database management

A total of 88,112 surgical procedures on the chest (including 38,999 procedures for primary lung cancer, 15,360 procedures for pneumothorax, and 4850 procedures for mediastinal tumor) were registered from 1000 CSUs to the NCD 2014 in the field of chest surgery. A total of 98.5 % of surgical procedures for a malignant pulmonary tumor, 95.5 % for a mediastinal tumor, and 93.3 % for bulla-related procedures were registered in the field of chest surgery, while quite a few surgical procedures for the pectus excavatum and palmer hyperhidrosis were registered in the other surgical fields (Fig. 4). Distribution of patient numbers according to each thoracic disease in the NCD for chest surgery was almost identical to the annual survey by JATS for the number of procedures categorized by thoracic disease (Fig. 5). The NCD had 4260 more registered procedures in comparison with the annual survey by JATS in 2014. More than 250 CSUs that had never provided data to the past annual survey registered data in the NCD. An Internet-based NCD in 2014 for chest surgery showed an inter-rater reliability of >94 % for each item (Fig. 6).

## Discussion

An NCD for chest surgery was organized based on the NCD system to provide an ideal database for chest surgery. A complete data-acquisition system linked with board certification for the second hierarchy of chest surgery and with accreditation of educational institutions for CSUs was successful and superior to the annual survey by JATS with respect to the number of registrations. The number of pulmonary resections for lung cancer registered in the NCD accounted for >95 % of all the cases registered in the Regional Bureau of Health and Welfare in Japan [4]. The system for NCD had a role as an eliminator of troublesome administrative procedures for chest surgeons and CSUs. This role may have been (at least in part), because JATS surveys are motivated by the intention to maintain institutional accreditation, whereas that of the NCD is by the individual qualification of application to the board of chest surgery. NCD can substitute for the annual survey by JATS in the future if data inputting can be more appropriate.

The registration system is dependent upon the field of surgery, because the NCD system is supported by JSS and nine other surgical societies. Procedures for pectus excavatum and palmar hyperhidrosis were not covered by the field of chest surgery. Pectus excavatum was mainly registered in the field of pediatric surgery for application to the board of pediatric surgery. The registration system should be unified regardless of fields of surgery in the future.

Lower accuracy tends to be a main problem for larger databases. Prevalence of inter-rater agreement through the audit system was satisfactory. In the future, Internet-based audit systems will be sustainable to maintain high quality in a large clinical database at reduced cost and reduced effort than site visits. The system will also be improved through greater experience and education on data input [5].

The goal of the NCD is to achieve high-quality surgical management [6]. To achieve this goal, the NCD will promote four sequential steps.

The first step is a risk model of pulmonary resection for lung cancer that is presented annually to each surgical unit for improving of surgical quality. Lung cancer is now a leading cause of death worldwide. Surgery remains the mainstay for complete cure, though other treatment modalities (e.g., molecular-targeted treatment or targeted radiation therapy) have been developed. Because of the aging society in Japan, many lung-cancer patients have comorbidities that can increase the risk of postoperative complications. The NCD can provide an annual estimated risk for preoperative status, surgical procedure, and oncologic status for each patient [7–14]. The NCD can be a “benchmark” for lung-cancer surgery with several risk factors. The program of quality improvement for management of lung cancer can be sustainable and can provide feedback to CSUs, thereby eliminating “cherry-picking” problems associated with other programs [4]. Updating the NCD for chest surgery can ensure that the latest methods in the surgical treatment of lung cancer are disseminated. Follow-up surveys will be linked to the NCD system to show the long-term outcomes after lung-cancer surgery. A total of 38,999 procedures for primary lung-cancer patients undertaken in 2014 were registered, which were considerably higher than the numbers in the general thoracic databases reported by the Society of Thoracic Surgeons and European Society of Thoracic Surgeons [15, 16] in terms of annual registration numbers. The impact on survival by surgical interventions will increase if the 5-year survival of these patients can be clarified by a follow-up survey.

The second step is a risk model for other thoracic diseases. Further expansion of data as oncologic data in relation to relatively rare thoracic diseases (e.g., thymic carcinoma, mesothelioma) will be registered to clarify the effects of surgery on survival.

The third step will be a nationwide program for improvement in health-care and evidence-based policies. Data can show regional disparities in surgery for chest diseases. Identification of surgeons by the license number registered by the JSS can show the surgeon’s performance for board certification but also their contribution to surgical outcomes [17]. Data on duration of hospitalization and operative costs (e.g., number of mechanical staplers) in relation to information on hospital discharge can aid cost-benefit analyses. Such research will aid recommendations for nationwide improvement of health-care and evidence-based policies. The government should support a further research project using the NCD.

The final step will be risk profiles compared with overseas databases. The NCD will be compared with those from the Society of Thoracic Surgeons and European Society of Thoracic Surgeons if collaboration can result in standardization of nomenclature and variables among these databases. Such comparisons can lead to the improvement programs of surgical quality worldwide.

In the conclusion, an NCD system for chest surgery was well organized with a high-registration rate and high-accurate inputting rate. This was thanks to the elimination of troublesome administrative procedures for chest surgeons at the time of application for board certification and for CSUs at the time of application for institutional accreditation through presentation of the annual survey by JATS, respectively. The system can sustainably provide important and up-to-date information relating to preoperative status, oncology, and best practice in Japan.
